# Lung metastasis of transitional cell cancer of the urothelium, with fungus ball-like shadows closely resembling aspergilloma: A case report and review of the literature

**DOI:** 10.3892/ol.2014.2076

**Published:** 2014-04-16

**Authors:** HIDEHIRO WATANABE, TOMONORI URUMA, TOKURO TSUNODA, GEN TAZAKI, ATSUSHI SUGA, YUSUKE NAKAMURA, SHUNSUKE YAMADA, TAKUMA TAJIRI

**Affiliations:** 1 Department of Respiratory Medicine and Infection Control, Tokyo Medical University, Ibaraki Medical Center, Ami, Ibaraki 300-0395, Japan; 2 Department of Respiratory Medicine, Tokai University Hachioji Hospital, Tokai University School of Medicine, Tokyo 192-0032, Japan; 3 Department of General Thoracic Surgery, Tokai University Hachioji Hospital, Tokai University School of Medicine, Tokyo 192-0032, Japan; 4 Department of Pathology, Tokai University Hachioji Hospital, Tokai University School of Medicine, Tokyo 192-0032, Japan

**Keywords:** air crescent sign, fungus ball, aspergilloma, metastatic lung cancer, transitional cell cancer

## Abstract

The present study reports the case of a 67-year-old female patient who was initially diagnosed with pulmonary aspergilloma. This diagnosis was based on a chest computed tomography (CT) scan showing a cavitary lesion of 3.5 cm in diameter, with fungus ball-like shadows inside, and an air crescent sign in the right upper lung. At 63 years old, the patient was treated for transitional cell cancer of the urothelium (non-invasive, pT1N0M0) by total cystectomy, ileal conduit diversion and urostomy. For 4 years post-operatively, the patient was healthy and had no clinical symptoms, and the air crescent sign was not identified by chest CT until the patient had reached 67 years of age. However, a final diagnosis of lung metastasis of transitional cell cancer of the urothelium was histopathologically identified subsequent to video-assisted thoracic surgery. Although it is rare that transitional cell cancer moves to the lung and makes a cavitary lesion, a differential diagnosis of cancer is necessary, even when examining infected patients with air crescent signs that are characteristic of aspergilloma. The physician must be mindful of metastatic pulmonary tumors that closely resemble aspergillomas, not only in infectious diseases, but also in oncological practice. Primary surgical removal should be considered.

## Introduction

Pulmonary aspergilloma is classified as non-invasive pulmonary aspergillosis, and is a chronic debilitating disease with clinical symptoms that include a chronic cough, slight fever and bloody sputum. However, a number of patients are asymptomatic (1). A series of typical chest computed tomography (CT) findings, including cavitary lesions with fungus ball-like shadows, air crescent signs, meniscus signs and double arches, are mostly caused by inflammatory lung diseases such as mycetoma, lung abscess, pulmonary tuberculosis and echinococcosis (2). These findings, typical for pulmonary aspergilloma, are frequently found in the upper lobes of the lungs (3). Imaging examinations are therefore considered to be an essential diagnostic tool for this condition. By contrast, it is quite rare that metastatic lung cancer makes a cavity lesion (4). Furthermore, a fungus ball-like structure is rarely found inside the lung cavity, particularly in transitional cell cancer (5). The present study reports a case of lung metastasis of transitional cell cancer of the urothelium in an asymptomatic patient who was initially diagnosed with pulmonary aspergilloma based on air crescent signs in the right upper lung. The patient provided written informed consent.

## Case report

### Patient history

The 67-year-old female patient of the present study had previously been diagnosed with transitional cell cancer of the urothelium (non-invasive, pT1N0M0), which had been treated with total cystectomy, ileal conduit diversion and urostomy at the age of 63. The patient had smoked 10 cigarettes a day between the ages of 30 and 60 and had then quit. The patient had no documented hypertension or diabetes mellitus. Subsequent to the finding of an abnormal shadow in the right upper lung, based on X-rays taken during the follow-up post-operative testing in April 2012, the patient visited the Department of Respiratory and Infection Control (Tokai University Hachioji Hospital, Tokyo, Japan) for a detailed examination. No such shadow had been detected in the lung during testing the previous year.

### Examination

**Figure 1:**
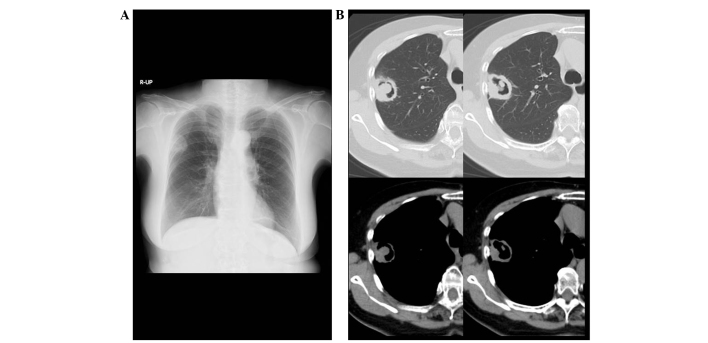
Initial chest X-ray and CT findings. (A) Plain chest X-ray showing a nodule 3.5 cm in diameter next to the pleura in the right upper lung. (B) Chest CT revealing a lung cavity with ball-like structures and air crescent signs. CT, computed tomography.

The patient’s blood pressure was 156/93, the heart rate was 79 bpm and regular, percutaneous oxygen saturation was 95% (room air) and there were no cardiopulmonary symptoms such as coughs and sputum. The patient had previously undergone a urostomy in the right lower abdomen. The blood tests, including those for tumor markers, were normal except for a slightly elevated white blood cell count. The aspergillus antigen and antibody were not present, and the β-D glucan level was also normal. The sputum culture detected no fungus or mycobacterial colonies throughout an 8-week incubation period. The chest CT showed a cavitary lesion that was 3.5 cm in diameter, with fungus ball-like shadows and air crescent signs next to the pleura in the right upper lung (Fig. 1). No nodular density was noted in the other lung fields. No pleural effusion or enlargement of the mediastinal lymph node was found. Based on the clinical and imaging findings, the patient was temporarily diagnosed with aspergilloma with a cavitary lesion and fungus ball-like shadows in the right upper lung.

### Treatment

**Figure 2:**
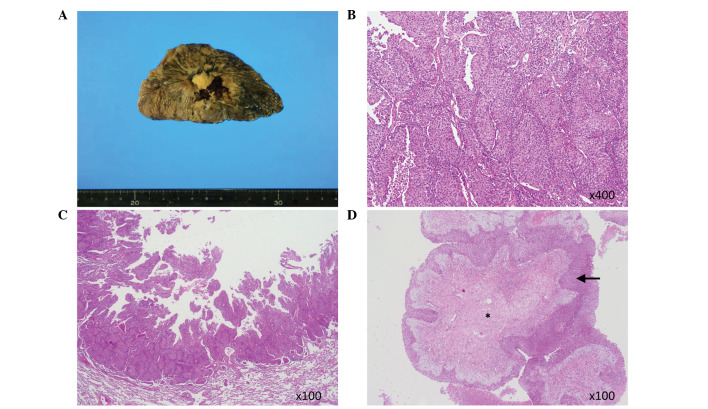
Resected lung tissue and histopathological findings (hematoxylin and eosin staining). (A) Resected lung tissue with fungus ball-like structures inside the cavity. (B) Planar growth of squamous cells in the surrounding area, mostly solid tumor with papillary growth progressing inward (magnification, ×100). (C) Transitional cell carcinoma (magnification, ×400). (D) Center of the fungus ball-like structure (magnification, ×100). Growth of transitional cell cancer (arrow) around the globular structure, covering the tumor stromal tissue (*) inside the cavity.

The primary treatment of aspergilloma is surgical removal, and the differentiation of a lung cancer is also required, therefore, video-assisted thoracic surgery was performed in May 2012, rather than a bronchoscopy, on the basis of the patient’s approval. The lesion was histopathologically identified as lung metastasis of transitional cell cancer of the urothelium (Fig. 2A). The histopathology confirmed that the tissues extended to the internal cavity wall and inner cavity, and that they were transitional cell cancer of the urothelium (Fig. 2B and C). The center of the fungus ball-like structure consisted of tumor stromal tissue covered with urothelial transitional cell cancer, not lung interstitial tissue (Fig. 2D). Mycetes, including *Aspergillus* sp., were not detected in the isolated tissue.

## Discussion

Pulmonary aspergilloma is caused by *Aspergillus* sp., a naturally existing fungus (with conidia of 2 to 4 μm in diameter). The fungus is inhaled and delivered to the abnormal lung cavities formed due to post-tuberculosis infection, pulmonary cysts, pulmonary fibrosis, open-chest surgery or dilated bronchi. The fungus then saprophytically proliferates and forms fungus balls. Aspergilloma typically affects residual cavities subsequent to lung tuberculosis and its complications are found in 11 to 17% of cases (6). Immunocompetent patients with aspergilloma are generally asymptomatic and usually aspergillus antigen-negative. Microscopic detection of the fungus in the sputum is difficult. Air crescent signs formed by the fungus balls in the cavitary lesion in the upper lung are typical (2), Isolated aspergilloma in a patient with no underlying disorders, such as the present case, should be primarily treated with curative surgical removal (7). Lung disorders with cavitary lesions requiring a differential diagnosis include lung tuberculosis, lung suppuration, pulmonary mycosis (aspergillosis), Wegener’s granulomatosis and primary lung cancer. However, the frequency of cavitation tumors in the lung is 2–5%; 2/3 to 4/5 of these are squamous cell cancer (8), whilst the remainder are adenocarcinoma (9). Possible mechanisms of tumor cavity formation include internal tissue necrosis, air trapping by the check valve, local extension by the elastic traction and bullae (10–13). However, there is never an apparent structure involved in lung cavities caused by these mechanisms.

In addition, pulmonary cavitation occurs in 4% of metastatic lung cancers (4). Pulmonary metastases of transitional cell carcinomas are normally found as solitary masses, multiple nodules or interstitial micronodules (14). Transitional cell cancer of the urothelium is only cited ~0.6% of the time as a cause of cavitary metastases (9,14–16). The present review of the literature classified differentiation of cavities with fungus ball-like structures into the following 3 categories: i) Cancer-associated cavitary lesions complicated with aspergilloma (17,18); for each case in the literature, the cavity was made with a primary lung adenocarcinoma, the mycotic infection happened internally and the fungus ball was created. ii) Lung cancer with cavitary lesions, including a fungus ball-like structure that was not a mycete (19–21); the studies reporting this witnessed fungus ball-like structures in cavities formed secondary to the internal necrosis of primary lung cancer, and the internal structure and the cavity-wall tissue had the same type of cancer cells, e.g., squamous cell carcinoma (19,20) or adenocarcinoma (21). However, these examples involved primary lung cancer, and not transitional cell cancer or metastatic cancer. iii) Pulmonary metastasis of transitional cell cancer; there are several studies in the literature on cavity formation associated with lung metastasis of transitional cell cancer of the urothelium (9,14–16,22–25). However, these are all studies of the cavity without fungus ball-like structures. Alexander *et al* (9) reported that the fungus ball-like structure appeared to be in the cavity at the time of the chest roentgenogram, but this was unclear. The present case involved lung metastasis of transitional cell cancer of the urothelium involving fungus ball-like structures in an isolated cavity with an air crescent sign, closely resembling aspergilloma. The histopathology confirmed that the tissue extended to the internal cavity wall and the inner cavity. Metastasis of transitional cell cancer with tumor stromal tissue around the primary lesion to the lung was indicated. There were no necrotic tissues or fungus ball-like structures in an intracavernous area. The central part of the fungus ball-like structure was tumor stromal tissue, and the surrounding tissue was a transitional cell carcinoma. The metastasized transitional cell cancer may have grown along the internal cavity wall, covered the later-growing tumor stromal tissue and formed the fungus ball-like structures in the cavity.

In conclusion, metastatic lung cancer that builds a fungus ball (aspergilloma)-like structure inside a cavity is quite rare. Since fungus balls/aspergilloma in an immunocompetent patient lack clinical symptoms and signs, a differential diagnosis of cancer and a surgical approach (26) will always be crucial for physicians.
